# Epidemiological profile of fungal keratitis in urban population of West Bengal, India

**DOI:** 10.4103/0974-620X.57310

**Published:** 2009

**Authors:** Suman Saha, Debdulal Banerjee, Archana Khetan, Jayangshu Sengupta

**Affiliations:** 1 Ocular Microbiology Division, Priyamvada Birla Aravind Eye Hospital, Kolkata, West Bengal, India; 2 Cornea and Ocular Surface Disease Clinic, Priyamvada Birla Aravind Eye Hospital, Kolkata, West Bengal, India; 3 Department of Microbiology, Vidyasagar University, West Midnapore, West Bengal, India

**Keywords:** *Candida* sp., fungal keratitis, therapeutic keratoplasty

## Abstract

**Background:**

Corneal diseases are one of the major causes of visual loss and blindness, second only to cataract. Amongst corneal diseases, microbial keratitis is a major blinding disease. In some countries, fungal keratitis accounts for almost 50% of patients with culture-proven microbial keratitis.

**Aim:**

This study was conducted to determine the epidemiological characteristics of fungal keratitis in an urban population of West Bengal and identify the specific pathogenic organisms.

**Methods:**

The charts of patients with microbial keratitis who attended the Cornea Services of Priyamvada Birla Aravind Eye Hospital from January to December 2008 were retrospectively reviewed. Records of patients with 10% KOH mount and culture positive fungal keratitis were analyzed for epidemiological features, laboratory findings and treatment outcomes.

**Results:**

Of the 289 patients of microbial keratitis included in the study, 110 patients (38.06%) were diagnosed with fungal keratitis (10% KOH mount positive). Of the 110 patients, 74 (67.27%) fitted the study inclusion criteria (10% KOH mount and culture positive). Forty five of 74 patients (60.81%) in the study group were in the older age group (>50 years). Ocular trauma in 35 cases (47.29%) was identified as a high risk factor and vegetative injuries in 17 cases (22.97%) were identified as a significant cause for fungal keratitis. Maximum organism source was from corneal scrapings in 41 cases (55%). The predominant fungal species isolated was *Aspergillus* sp (55.40%) followed by *Candida albicans* 14 cases (18.91%) and *Fusarium* sp. in 8 cases (10.81%). Agricultural activity related ocular trauma was the principal cause of mycotic keratitis and males were more commonly affected. Thirty of 74 cases (40.55%) of the culture positive patients healed with corneal scar formation with medical treatment whereas 44 cases (59.45%) required therapeutic keratoplasty.

**Conclusion:**

Fungal keratitis is an important cause of microbial keratitis with injury to the cornea being a leading predisposing factor. Although *Aspergillus* sp. was implicated in most of the patients in our study population, *Candida* sp. were found in higher numbers than previously reported. Keratitis caused by filamentous fungi responds adequately to medical management. Therapeutic keratoplasty continues to remain an important treatment modality in infections with *Candida* sp. Early diagnosis with prompt identification of the pathogenic organism is mandatory to initiate appropriate therapy and thereby reduce morbidity.

## Introduction

Corneal blindness is a major health problem throughout the world.[1] According to the World Health Organization report, it is estimated that ocular trauma and corneal ulceration result in 1.5 to 2 million new patients of corneal blindness annually, posing a major public health problem for developing countries.[2] The etiological and epidemiological pattern of corneal ulceration varies significantly with patient population, geographical region and prevailing socioeconomic conditions.[3] Srinivasan *et al* from South India reported that 44% of all central corneal ulcers were caused by fungi.[4] More than 70 species of filamentous fungi have been identified as the etiological agents of fungal keratitis.[5] Early diagnosis of fungal keratitis and its treatment is important in preventing complications and loss of vision.

We conducted this study to identify the epidemiological features, laboratory findings and treatment outcomes amongst patients attending the Cornea service of a tertiary eye care hospital in Kolkata, India.

## Materials and Methods

After obtaining approval from the institutional review board, the medical records of 289 clinically diagnosed patients of microbial keratitis, who attended Cornea service of Priyamvada Birla Aravind Eye Hospital, a tertiary eye care hospital in Kolkata, India, from January to December 2008, following the introduction of in-house microbiology services, were evaluated retrospectively for duration of symptoms, predisposing factors like trauma, associated ocular conditions, any systemic diseases, therapy received before presentation, history of corticosteroids use and previous eye surgery.

The Cornea service protocol for management of patients with a clinical diagnosis of microbial keratitis was identical for all patients in this study. Corneal scraping was performed in all patients by an ophthalmologist using a sterile #15 Bard Parker surgical blade, following the instillation of local anesthetic eye drop such as 0.5% proparacaine, under aseptic conditions. The material was obtained from the active margin and base of the ulcer following debridement of superficial mucus. In patients with deep corneal infiltrates with or without anterior chamber exudates, a deep scraping, corneal biopsy or anterior chamber tap was additionally performed. In patients where therapeutic keratoplasty was performed, corneal scraping was repeated at the time of surgery, and excised corneal buttons were subjected to microbiological and histopathological procedures.

The material obtained was directly smeared on a labeled slide for 10% KOH wet mount, Gram′s staining[5,6] and incubated directly in solid media (10% sheep blood agar, Sabourand′s Dextrose agar, potato Dextrose agar, nutrient agar and chocolate agar) in a row of C-shaped streaks. Brain heart infusion broth was used for a few patients in whom fungal keratitis was strongly suspected. The culture plates (10% sheep blood agar plate, chocolate agar and brain heart infusion broth) were kept for 10 days at 37°C, and discarded if no growth was obtained and no turbidity was seen after 10 days. The SDA plates were observed for 21 days in room temperature. Any growth obtained was further identified by microscopic aspects of texture, pigmentation, mycelium arrangement and conidium types by lactophenol cotton blue mount. Yeast was differentiated by germ tube formation and sporulation on cepakdox medium. The culture was considered positive if the growth of the same fungal species was found in more than one solid media.

Patients with a diagnosis of fungal keratitis as determined by positive microbiology result (patients fungal elements identified in 10% potassium hydroxide (KOH) wet mount preparation and gram staining, and positive growth for same fungus in at least two solid media) were identified.

Epidemiologic and clinical data were recorded.

Two modes of treatment were identified from the case records.

- Topical therapy with natamycin eye drops (5%), amphotericin B (0.15%) and prepared 2% voriconazole eye drops.
- Therapeutic penetrating keratoplasty.

Patients undergoing topical therapy were started on either natamycin or voriconazole depending upon the choice of the physician. Identification of *Candida* species in culture or worsening of ulcer to topical monotherapy led to initiation of therapy with Amphotericin B eye drops. All patients received oral ketoconazole as adjunctive therapy. Depending on the severity of keratitis at presentation, topical or surgical therapy was initiated. Decision for therapeutic keratoplasty was also taken in case of non-responsiveness or worsening (increase in size of infiltrate/hypopyon, extension to the limbus, corneal melting) of condition despite maximal medical therapy.

## Results

**Figure 1:**
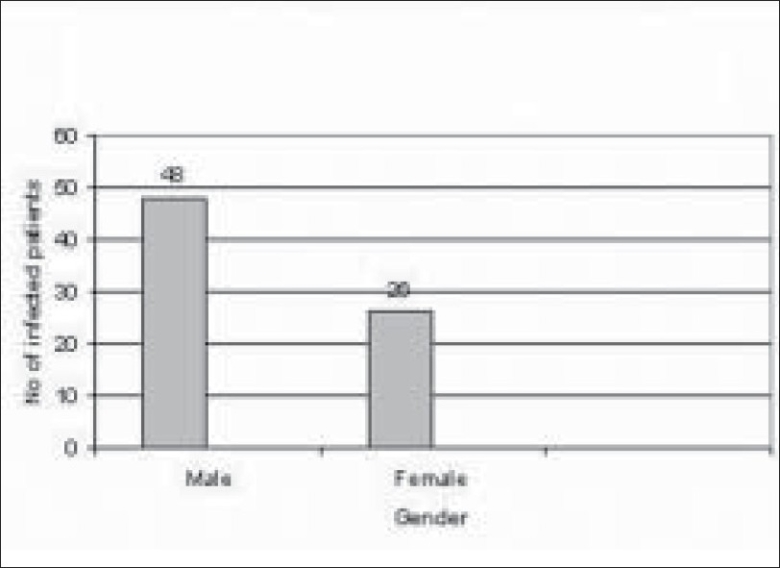
Distribution of keratitis between male and female patients

**Figure 2:**
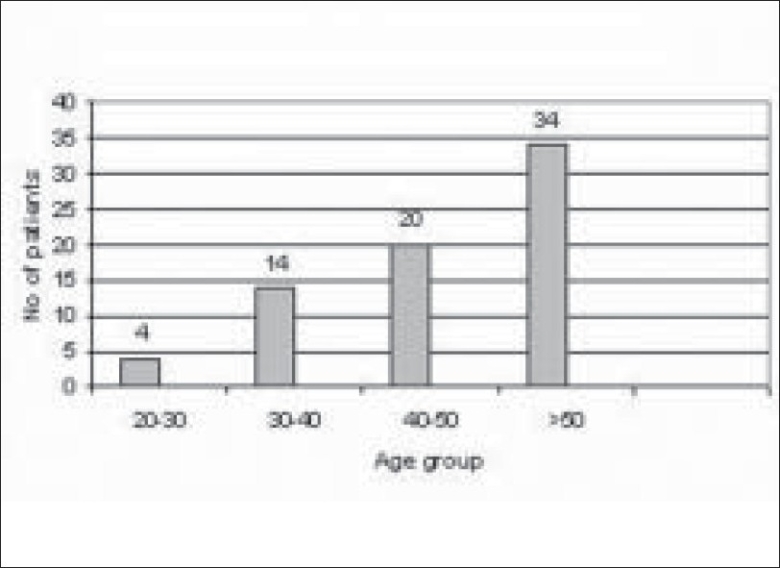
Age distribution of keratitis patients

**Figure 3:**
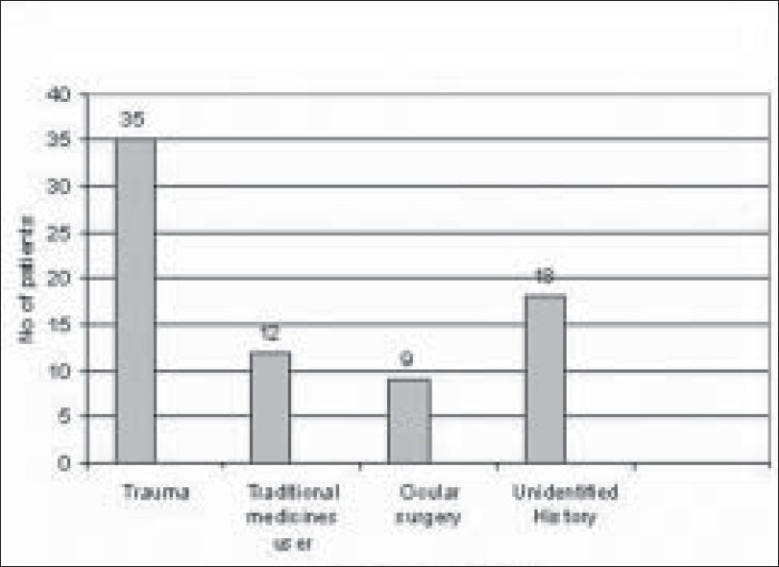
Types of trauma responsible for fungal keratitis

**Figure 4:**
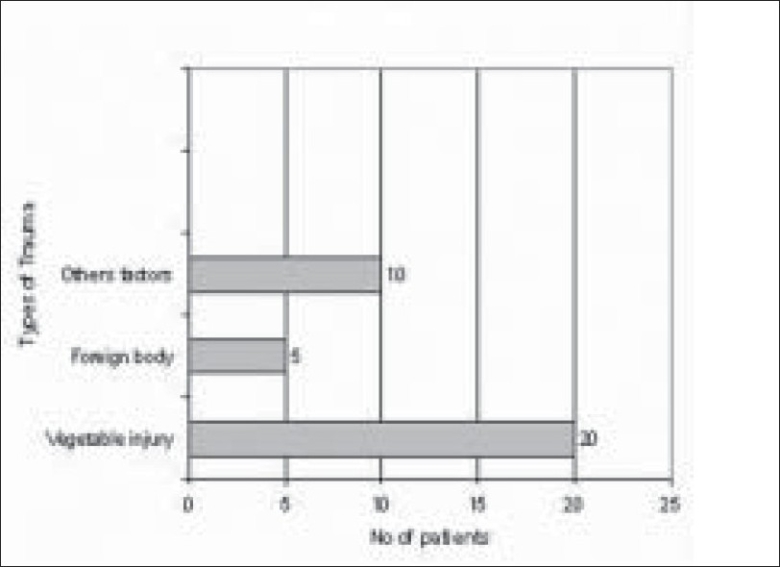
Predisposing factors for keratitis

**Figure 5:**
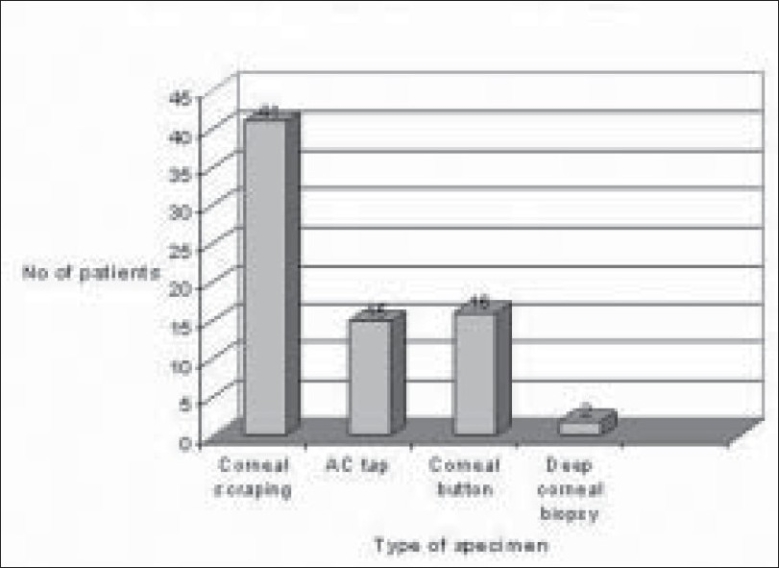
Organism source of keratitis

**Figure 6:**
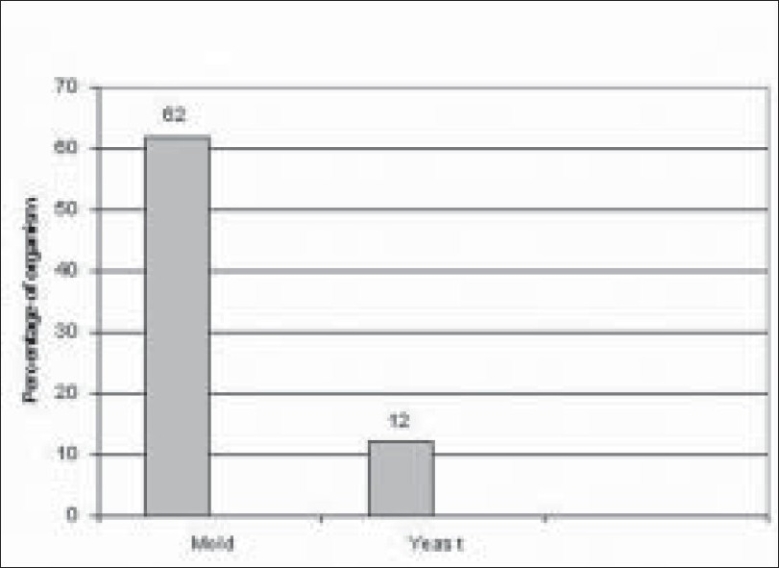
Mold and yeast distribution

**Figure 7:**
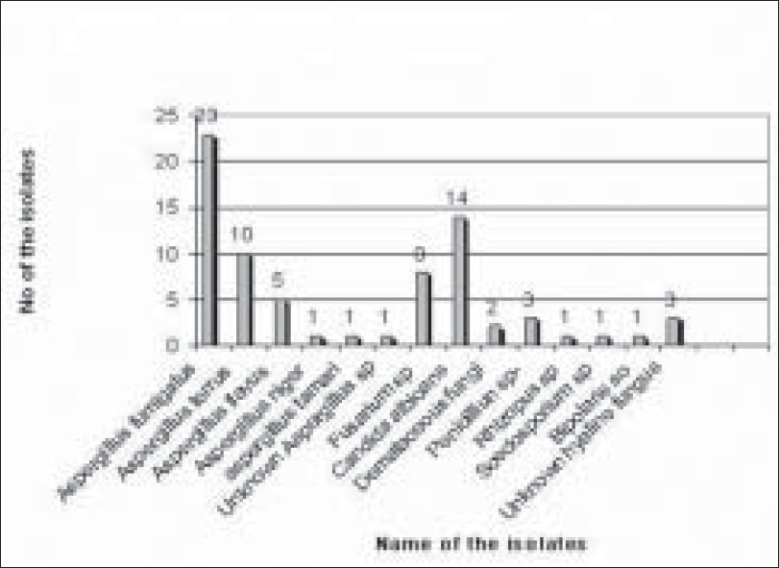
Percentage of mold and yeast species isolated from keratitis

Of 289 microbial keratitis patients, 110 patients (38.06%) were diagnosed as fungal keratitis based on clinical findings as well as observation of fungal filaments in KOH mount and Grams staining. Of these patients, 74 patients (67.27%) were culture positive fungal keratitis; the remaining 36 (32.73%) patients were only smear positive. There were more male patients (48/74 i.e. 65%; 95% CI 53–74) compared to female patients (26/74 i.e. 35%; 95% CI 26–46) in our study. The male to female ratio was (1 : 1.84) [Figure 1]. The mean patient age was 53 years. Older patients (>50 years) were more frequently affected (34/74 i.e. 46%; 95% CI 35–57) [Figure 2]. There was a history of trauma in 35 patients (48%; 95% CI 36–58) followed by history of steroid usage in 12 patients (16%) and previous ocular surgery in 9 patients (12%). No obvious predisposing factor could be identified in 17 patients (23%) [Figure 3]. Among patients with a history of injury, trauma with vegetative matter was found in 20 patients [Figure 4]. Maximum culture positivity was found from corneal scraping, 41 of 74 patients (55%; 95% CI 44–66), followed by recipient corneal button obtained during therapeutic keratoplasty, 16 of 74 patients (22%; 95% CI 12–30), anterior chamber tap 15 of 74 patients (20%; 95% CI 13–32) and deep corneal biopsy in 2 patients (3%; 95% CI 0–9) [Figure 5]. Of fungal isolates, 62 out of 74 isolates; (83.8%) were molds and 14 isolates (18.91%) were yeasts [Figure 6]. *Aspergillus* species was identified as the most common etiologic agent (41 of 74 isolates; 55.4%) followed by *Candida albicans* (14 isolates; 18.91%). *Fusarium* species, *Dematiaceous* fungi, *Penicillium* sp., *Rhizopus* sp., *Scedosporium* sp., *Bipolaris* sp. and many unidentifiable fungal species were isolated from the fungal keratitis patients [Figure 7]. Thirty (40.55%) patients with culture positive fungal keratitis patients showed response to topical medical therapy with the formation of corneal scar. The remaining 44 patients (59.45%) of mycotic keratitis patients needed therapeutic keratoplasty. This included 16 cases of 41 *Aspergillus* keratitis (39.02%) and 5 cases of 8 (62.5%) of *Fusarium* keratitis. In our study all patients with *Candida* sp. infections required a surgical intervention. Ten patients among 74 (7.4%) culture positive fungal keratitis cases need immediate therapeutic keratoplasty at presentation.

## Discussion

Our study revealed that fungal keratitis accounted for 38.06% of the total microbial keratitis patients who presented to our center. This high prevalence of fungal pathogens in East India was not so different from that found in similar studies in Bangladesh (36%), Ghana (37.6%) and South Florida (35%).[7,8]

Injury to the cornea was the leading cause of fungal keratitis in our study (35 patients; 48%). A history of corneal trauma with vegetable matter or organic matter has been reported in 55–65% of fungal keratitis.[4,5,9] However, a study from the Northern United States reported trauma as the inciting event in only 8.3% of patients.[10] Steroid use as initial therapy has been reported in 1–30% of patients having microbial keratitis.[4,5,9,11] In our study, 12 patients (16%) had history of steroid usage.

Direct examination of smears from corneal scrapings examined by 10% KOH wet mount preparation (95%) and or gram stain (85.22%) continue to be an important mode of identifying causative organism in patients with microbial keratitis. Other methods include anterior chamber tap, corneal biopsy and examination of corneal buttons from patients undergoing therapeutic keratoplasty. The sensitivity of KOH and gram stain in preliminary identification of fungal filaments has been substantiated in other studies.[5,7,12,13] In KOH mount slide, fungal filaments appear as refractile hyphae with septate or aseptate, branching or non-branching filaments. Some filaments look brown due to melanin pigments in some species of fungi. On the other hand, yeast cells are oval or round and colorless and sometimes produce pseudohyphae in the KOH wet mount preparation.

*Aspergillus* species (55.4%) and *Candida* species (18.91%) were found to be the major etiologic agents of fungal keratitis in this study followed by *Fusarium* sp. (10.81%). Other studies have implicated *Fusarium* species (37–62%) and *Aspergillus* species (24–30%) as major pathogens with Dematiaceous fungi as the cause of 8–16.7% of patients with fungal keratitis.[5,12] In India, *Aspergillus* sp. is the main etiological organism responsible for mycotic keratitis followed by *Fusarium* sp. except in South India where *Fusarium* keratitis was maximum to up to 43%.[5] In Nepal Upadhyay *et al*[7] found that *Aspergillus* sp. accounted for 47% of all fungal pathogens followed by *Candida* sp. (13.2%) and *Fusarium* sp. (11.7%). Gopinathan *et al*[11] from India have reported *Candida* sp. as a rare fungal corneal pathogen (0.7%). In another series of 24 patients from Wills Eye Hospital, Philadelphia, *Candida* sp. was identified in 45.8% of patients of fungal keratitis.[10] The relatively high isolation of *Candida* sp. in this set of patients deserves attention. It has been reported that *Candida* infection is more common in temperate climate of the West compared to the tropical climate of East. However, it is also known that the growth rate of yeast is maximum in warm and moist condition.[14] Basak *et al*[15] reported 1.1% incidence of *Candida* positive patients among 509 mycotic keratitis patients. This study was conducted in a hospital with a rural population base. Factors other than climatic conditions, such as altered local defense mechanisms and immune suppression may also be responsible for the higher incidence of *Candida* infection in some studies compared to others.

Surgical intervention in the form of therapeutic keratoplasty continues to be an important mode of management. In our study, 40.55% patients showed response to medical therapy, while 59.45% of mycotic keratitis patients including 39.02% of *Aspergillus* keratitis and 60% of *Fusarium* keratitis needed therapeutic keratoplasty. Regina L *et al* reported from Texas that out of 29 *Candida* keratitis patients, 15 patient (51.72%) required surgical intervention of which 13 patient had therapeutic penetrating keratoplasty and 2 eyes needed enucleation.[16] Vemuganti *et al* reported that maximum fungal species identified from corneal buttons after therapeutic keratoplasty were *Fusarium* sp. in 30 (39%) and *Aspergillus* sp in 25 (33%) buttons.[17] In our study it was seen that all patients suffering from *Candida*, *Rhizopus*, *Bipolaris* and *Scedosporium* species infections required a surgical intervention.

To summarize, our study addresses the profile of fungal pathogens responsible for corneal ulceration among the urban population of West Bengal. While *Aspergillus* sp. remains the most predominant organism, *Candida* sp. were isolated in high numbers. Topical therapy is not always sufficient to eradicate infection in patients with fungal keratitis irrespective of the identified organism though *Aspergillus* and *Fusarium* infections seem to show some response to medical management. Therapeutic keratoplasty continues to remain an important treatment modality particularly in infections with *Candida* sp. Particular care should be taken in identification of the pathogenic organism with special stress on basic microbiological procedures like KOH and Gram staining to initiate appropriate therapy.
